# Stigma and discrimination in sexually diverse nursing students: a qualitative study

**DOI:** 10.15649/cuidarte.4043

**Published:** 2024-11-13

**Authors:** Bertha Lucía Correa-Uribe, Leidy Johanna Rueda-Díaz

**Affiliations:** 1 Universidad Industrial de Santander, Bucaramanga, Colombia. E-mail: blcorrea@uis.edu.co Universidad Industrial de Santander Universidad Industrial de Santander Bucaramanga Colombia blcorrea@uis.edu.co; 2 Universidad Industrial de Santander, Bucaramanga, Colombia. E-mail: ljruedad@uis.edu.co Universidad Industrial de Santander Universidad Industrial de Santander Bucaramanga Colombia ljruedad@uis.edu.co

**Keywords:** Students, Nursing, Social Stigma, Social Discrimination, Gender Diversity, Nursing, Estudiantes de Enfermería, Estigma Social, Discriminación Social, Diversidad de Género, Enfermería, Estudantes de Enfermagem, Estigma Social, Discriminação Social, Diversidade de Gênero, Enfermagem

## Abstract

**Introduction:**

Sexually diverse nursing students often face situations of stigma and discrimination within the university environment, which can significantly affect their educational experience and well-being.

**Objective:**

To understand nursing students' experiences regarding stigma and discrimination due to their sexual orientation or gender identity during their academic training.

**Materials and Methods:**

A qualitative phenomenological approach was employed. Semi-structured interviews were conducted with eight nursing students who identify as part of the LGBTQIA+ community. The collected data were analyzed using thematic analysis with the assistance of ATLAS.ti software.

**Results:**

The participants were aged 20 to 22 years and were in their sixth to tenth academic semester. Data analysis identified three categories: stigma and discrimination, coping strategies, and university experience.

**Discussion:**

The university community, recipients of care, and workers at practicum sites display stigmatizing and discriminatory behaviors toward nursing students who are sexually diverse and break social paradigms of what is considered normal.

**Conclusion:**

Nursing students face stigma and discrimination based on their sexual orientation or gender identity during their academic training. To navigate these situations, they adopt coping strategies ranging from concealing their identity to reclaiming words insulting. The university is perceived as a safe space for expression and personal growth.

## Resumen.

**Introducción:**

Los estudiantes de enfermería sexualmente diversos a menudo enfrentan situaciones de estigma y discriminación dentro del ámbito universitario, lo que puede afectar significativamente su experiencia educativa y bienestar.

**Objetivo:**

Comprender la experiencia de estudiantes de enfermería frente al estigma y discriminación por su orientación sexual o su identidad de género en su formación académica.

**Materiales y Métodos:**

Se empleó un enfoque cualitativo fenomenológico. Se realizaron entrevistas semiestructuradas a ocho estudiantes de enfermería que se identifican como parte de la comunidad LGBTQIA+. Los datos recopilados fueron analizados mediante análisis temático utilizando el software ATLAS.ti.

**Resultados:**

Los participantes tenían edades comprendidas entre 20 y 22 años, y cursaban entre sexto y décimo semestre académico. El análisis de los datos permitió identificar tres categorías: estigma y discriminación, estrategias de afrontamiento y experiencia universitaria.

**Discusión:**

La comunidad universitaria, los sujetos de cuidado y los trabajadores de los sitios de prácticas formativas muestran conductas estigmatizantes y discriminatorias hacia aquellos estudiantes de enfermería sexualmente diversos que rompen con los paradigmas sociales de lo que se considera normal.

**Conclusión:**

Los estudiantes de enfermería enfrentan estigma y discriminación por su orientación sexual o identidad de género en su formación académica. Para manejar estas situaciones, emplean estrategias de afrontamiento que abarcan desde el ocultamiento de su identidad hasta la reapropiación de insultos. La universidad se presenta como un espacio seguro para la expresión y crecimiento personal.

## Resumo.

**Introdução:**

Os estudantes de enfermagem com diversidade sexual frequentemente enfrentam situações de estigma e discriminação no ambiente universitário, o que pode afetar significativamente sua experiência educacional e seu bem-estar.

**Objetivo:**

compreender a experiência de estigma e discriminação dos estudantes de enfermagem com base em sua orientação sexual ou identidade de gênero em sua formação acadêmica.

**Materiais e Métodos:**

Foi realizado um estudo de desenho qualitativo fenomenológico. Foram realizadas entrevistas semiestruturadas com oito estudantes de enfermagem que se identificam como parte da comunidade LGBTQIA+. Os dados coletados foram analisados por análise temática usando o software ATLAS.ti.

**Resultados:**

A idade dos participantes variou de 20 a 22 anos e eles estavam no sexto ao décimo semestre acadêmico. A análise dos dados identificou três categorias: estigma e discriminação, estratégias de enfrentamento e experiência universitária.

**Discussão:**

A comunidade universitária, os sujeitos do cuidado e os trabalhadores nos locais de prática de treinamento demonstram comportamentos estigmatizantes e discriminatórios em relação a estudantes de enfermagem sexualmente diversos que rompem com os paradigmas sociais do que é considerado normal.

**Conclusão:**

Os estudantes de enfermagem enfrentam estigma e discriminação com base em sua orientação sexual ou identidade de gênero em seu treinamento acadêmico. Para lidar com essas situações, eles empregam estratégias de enfrentamento que vão desde esconder sua identidade até se reapropriar de insultos. A universidade é apresentada como um espaço seguro para a autoexpressão e o crescimento pessoal.

## Introduction

Sexual diversity is a fundamental aspect of human identity, but unfortunately, LGBTQIA+ youth often face significant challenges related to their sexual orientation and gender identity. In this context, higher education institutions become scenarios where these challenges can be heightened. Sexually diverse students are at risk of experiencing stigma and discrimination in this academic environment^1^^, ^^2^.

Stigma is a dynamic process in which individuals and structures interact continuously, influenced by power, control, and domination^3^. Within stigmatization processes, four interrelated types have been identified: enacted stigma, felt (or perceived) stigma, self-stigma (or internalized stigma), and anticipated stigma^4^. Enacted stigma is seen in the discrimination or unfair treatment of a stigmatized group, based on negative beliefs and feelings prevalent in society and culture. Felt stigma describes how a stigmatized group perceives injustice or discrimination related to their condition. Self-stigma occurs when individuals assume and accept their stigmatized condition. Finally, anticipated stigma refers to concerns about facing injustice or discrimination because of their condition. Discrimination, conversely, is manifested through rejection behaviors that violate the rights of stigmatized individuals.

Exposure to stigma and discrimination is strongly associated with psychological distress^5^ and often triggers adverse health outcomes and risk behaviors^6^ such as anxiety, depression^7^, self-harm, and suicidal ideation^7^^, ^^9^. It can also cause problems in the areas of work, study, leisure, and interpersonal relationships^4^.

In the university setting, LGBTQIA+ students report experiences of stigma and discrimination from the academic community^10^^, ^^11^, including heterosexual students, administrative personnel, and faculty. The latter may abuse their power and not promote inclusive educational practices. Abuse of power translates into negative academic leadership, imposition of clearly heteronormative relationships in the classroom, discrimination, and contempt for students who deviate from this pattern, which affects students academically and emotionally^12^. This study focused on a particularly vulnerable group: nursing students of diverse sexual orientations. It has been observed that these students, in challenging normative paradigms, continue to face stigma and discrimination both in the classroom and in practicum settings. Despite the apparent need to address this issue, no studies were found that report on the experiences of LGBTQIA+ students enrolled in nursing programs regarding their sexual orientation or gender identity. Consequently, significant gaps persist in the research on how stigma and discrimination towards these students are shown and the role of educational institutions in perpetuating these attitudes. A thorough understanding of these experiences is important to develop practical recommendations that will improve the well-being of these students and transform their future academic experiences.

In this context, the present study aimed to understand nursing students' experiences of stigma and discrimination based on sexual orientation or gender identity in their academic training.

## Materials and Methods

The Standards for Reporting Qualitative Research (SRQR)^13^ recommendations were followed in preparing the report of this study. A qualitative, phenomenological study design was conducted.

Phenomenology explores the experiential realities that are not very communicable but are fundamental to understanding the psychic life of each individual^14^.

This study's population was LGBTQIA+ nursing students enrolled in a public university's nursing program in Bucaramanga. The curriculum lasts ten academic semesters and includes practicums in community and clinical settings beginning in the third semester.

Due to the nature of the study, the number of participants was not pre-determined; however, the sample size was determined by data saturation. Inclusion criteria for participants were to be 18 years of age or older, to self-identify as gay, lesbian, bisexual, transgender, transsexual, questioning, or any other sexual orientation or gender identity that does not match the sex assigned at birth, and to be enrolled in the nursing program. The sample was recruited using snowball sampling. This sampling method is often used to recruit hard-to-reach participants^15^. This method was also chosen because there are no clear and direct channels for recruiting enrolled LGBTQIA+ students and because the literature suggests that LGBTQIA+ individuals have support networks that include other LGBTQIA+ individuals^16^. First, we reached out directly to a student who had come out as part of the LGBTQIA+ community to the program's faculty. In addition to being interviewed for the study, this student shared information about the research with other students of diverse sexual orientation/gender identity and provided telephone contact for at least two other students interested in participating. This approach allowed the research team to contact new participants directly, continuing the snowball recruitment process.

Data were collected between February and June 2023 through semi-structured interviews conducted in a single session per participant. The guiding question was “What have been your experiences with stigma and discrimination due to your sexual orientation or gender identity during your academic training?” and further questions were developed from this to allow the interviewee to delve deeper into the topic. Questions may include, but are not limited to, the following: During your nursing education, have you ever been stigmatized or discriminated against because of your gender identity or sexual preference? By whom? How have you responded to situations of stigma and discrimination? The interviews were conducted in a private place at the Faculty of Health of the Universidad Industrial de Santander and were conducted and recorded by one of the researchers. On average, each interview lasted one hour. The researcher who interviewed the participants had at least 20 years of experience in conducting semi-structured interviews.

All interviews were transcribed verbatim by another researcher. Once transcribed, each interview was carefully read to capture the essence of each experience reported. The information was collected and recontextualized using an open coding system with ATLAS.ti software. The interpretation process involved copying, intensive reading, note-taking, analysis, a first draft of the report, initial coding, grouping, and determining categories and subcategories. We clarify that after the initial coding of the data using the software, we proceeded with a detailed analysis of the frequencies of the codes that emerged from the semi-structured interviews. This process involved identifying and counting occurrences of relevant codes that captured experiences of stigma and discrimination based on sexual orientation or gender identity among LGBTQIA+ nursing students. The most frequent codes were grouped into main thematic categories, which were refined through iterative discussion among the researchers. To ensure the scientific rigor of the research, the criteria of credibility, confirmability, dependability, and transferability were applied^17^.

To ensure credibility, verbatim transcripts of the interviews were used, and the findings and interpretations were discussed with four of the study participants. Confirmability was ensured through a faithful analysis of the transcripts based on the interview recordings. Dependability was fostered through independent coding of the data by the researchers and an external expert, who reached a general agreement on the findings. Finally, transferability was addressed by providing a detailed description of the participants and the context in which the study occurred. The collected data are available for free access and consultation in Mendeley Data^18^.

The present study was considered minimal-risk research according to Resolution 008430 of 1993 of the Colombian Ministry of Health^19^ and was approved by the Ethics Committee for Scientific Research (CEINCI, for its acronym in Spanish) of the Universidad Industrial de Santander. Participation of the nursing students was voluntary, and all signed informed consent prior to the interviews. It was ensured that all data collected were kept completely anonymous.

## Results

Eight interviews were conducted and analyzed with LGBTQIA+ nursing students between the ages of 20 and 22 who were in their sixth to tenth semesters of the nursing program. All participants had already had internships in clinical and community settings. Descriptions of the participants are shown in Table 1.

**Table 1:** Characteristics of participants. Bucaramanga, 2023

Code	Sexual orientation	Age (years)	Semester
E1	Lesbian	22	Eighth
E2	Lesbian	21	Eighth
E3	Lesbian	21	Seventh
E4	Bisexual	22	Eighth
E5	Homosexual	22	Tenth
E6	Fluid	21	Sixth
E7	Homosexual	20	Sixth
E8	Homosexual	20	Sixth

In the analysis process, three categories emerged, grouped as follows: I. Stigma and discrimination II. Coping strategies III. University experience. As shown in Table 2, for the category "stigma and discrimination," the most frequent deductive codes are "negative attitudes and prejudices" (75.00%), and the emergent codes are "derogatory comments" (62.50%). In "coping strategies," the deductive codes include "identity concealment" (75.00%), and the emergent codes are "stories of concealment and submission" (62.50%). In "university experience," deductive codes such as "university as a safe haven" have a frequency of 62.50%, while emergent codes such as "space for freedom" appear with a frequency of 37.50%.

### Stigma and discrimination

This category encompasses the experiences of nursing students with diverse sexual orientations in relation to stigma and discrimination within the academic environment. The stories reveal various ways these students face prejudice and negative attitudes from peers, professors, and other members of the university community.

**Table 2:** Physical Activity Questionnaire for Adolescents (PAQ-A) by type of school.

Category	Deductive codes	Frequency (n=8)	Emergent codes	Frequency (n=8)
Stigma and discrimination	Negative attitudes and prejudices	6 (75.00%)	Derogatory comments	5 (62.50%)
	Traditional gender expectations	5 (62.50%)	Stereotypical perceptions	3 (37.50%)
	Gender roles in the nursing profession	5 (62.50%)	Perceptions of non discrimination among women	2 (25.00%)
	Negative nonverbal behaviors Rejection	4 (50.00%) 4 (50.00%)	Observations of negative behaviors	3 (37.50%)
Coping strategies	Identity concealment	6 (75.00%)	Stories of concealment and submission	5 (62.50%)
	Submission	5 (62.50%)	Ignoring derogatory comments	1 (12.50)
	Positive assertion of identity	4 (50.00%)	Reclaiming insults	3 (37.50%)
University experience	University as a safe haven	5 (62.50%)	Space for freedom	3 (37.50%)
	Self-acceptance and free expression	5 (62.50%)	Personal growth	1 (12.50%)
	Broadening perspectives	4 (50.00%)		
	Personal development	5 (62.50%)		

### Stigma based on sexual orientation

In the academic environment, some classmates and professors use derogatory words or comments that reveal negative attitudes and prejudices about the interviewees' sexual orientation.

*"I told the client my full name, where I was from, my practicum semester, and explained the procedure I was about to perform. The client agreed to let me proceed with the cytology. However, a classmate who was present made an extremely inappropriate comment that made me feel bad. It was something along the lines of, 'Ma'am, don't worry anyway, he's a faggot.'" (E5) "I have a classmate and he has said it directly to me. I mean, I make a comment and he is like: 'Oh, shut up, you're a lesbian'." (E3) "The professors in one of the courses tend to make fun of us a lot. They say things like, 'Oh, are you gay or something? You seem a gay...'" (E4)*

*.*

### Gender stereotypes

The accounts demonstrated stereotypical and discriminatory perceptions by linking their physical appearance to gender stereotypes and expressing disapproval of non-normative gender manifestations.

One student shared,

"*A practicum instructor once told me, 'You seem to have the strength of a boy, and since there are no boys in this group, you're useful to help me move this person.'" (E1) The same student mentioned that nursing assistants at a practicum site have told her, 'You're big, you don't look feminine-you must be part of the [LGBTQIA+] community'"*

*.* Another student shared experiences of discrimination based on the use of accessories considered feminine, such as earrings, and how this provoked negative reactions:

*I was wearing my earrings, and a classmate was saying something like, 'Oh, girl, I don't know what...' Then the porter overheard and said to me, 'You look really masculine with those earrings, don't you?' He looked at me with such hatred, like I'd done the worst thing in the world to him. I was just like, 'What?' I mean, I'm just existing.'"(E6)*

Another student also reported experiences of discrimination, including from patients' relatives, demonstrating the persistence of gender stereotypes in practicum settings:

*"It was a family member of a patient who had suffered a head trauma. I was providing wound care for the patient when his wife noticed my earrings. She asked me why I was wearing them, saying earrings were for women. She also gave me a dirty look, as if she didn't want me there taking care of her husband." (E7)*

On the other hand, one student did not identify having experienced any form of discrimination:

*"I don't consider that at some point I have been discriminated against because with women, it is not so evident, maybe, sometimes, those sexual preferences." (E2)*

*.* The latter account suggests that women's experiences of sexual orientation may be mediated by gender stereotypes, which may make such experiences less visible or viewed differently than their male counterparts.

### Attitudes and prejudices of the university community

The experience of discrimination based on sexual orientation or gender expression is palpable among peers, faculty, and other members of the university community. These attitudes are manifested through nonverbal behaviors and negative comments that reveal pre-existing prejudices:

*"The attitude of more classmates and professors, like, they roll their eyes at you, they show signs of annoyance. I have seen it during practicum, when they see you... I don't know how to explain it... I mean... you already know that they have you in their sights and that the professors already know who we are" (E3). "When there is news related to aggressions against LGBTQIA+ people, I have heard students and professors make comments like, 'See? Who sends them to show what they are, that's why they got kill.'" (E1) "I feel those nasty gestures or comments in class, like when they say, 'Split up those girls over there, split up those people over there, split up.' It's an obvious rejection." (E4)*

*.*

### Coping strategies

Two categories of coping mechanisms were identified that enable students to navigate the various scenarios of their academic lives: personal protection and empowerment.

### Personal protection strategies

This strategy refers to actions the students take to avoid or minimize exposure to potentially discriminatory or stigmatizing situations. Concealment, submission, and disregard all into this category.

Concealment consists of modifying aspects of identity or behavior.

*"Normally, you try to hide certain attitudes in certain places, for example, with my friends, with certain professors, with certain people. You know you can't be that affectionate because what are they going to say? What if they say something? Well, we're not in a position to get into a quarrel with somebody." (E1)*

In another case, the student chooses not to disclose to people he perceives as homophobic his sexual orientation and chooses to hide it or give no indication of it.

*"The mechanism that I use to avoid a homophobic person is to avoid telling them that I am homosexual or to avoid implying anything like that." (E5)*

*.*

Another student's account demonstrates the use of this strategy by deliberately adopting a serious demeanor and modifying his behavior in certain contexts in an effort to hide aspects of his identity or avoid drawing attention to himself. Another student's account demonstrates the use of this strategy by deliberately adopting a serious demeanor and modifying his behavior in certain contexts in an effort to hide aspects of his identity or avoid drawing attention to himself.

*“I always keep a serious demeanor. Because the professor is someone who is evaluating me and just because I'm gay, she could drop my grades to rock bottom. So, I’m like, 'Here, no, don’t say anything. Outside, yes.' That happened to me with her [a professor]. You don't know how she’s going to react, so you become a bit self-conscious. Yes, you do become a little self-conscious about how you behave." (E6)*

Submission and disregard strategies are activated in response to situations of stigma or discrimination that have already occurred or are ongoing. Submission involves conforming to the expectations or demands of others, often to avoid conflict or further discrimination. It is based on deep-rooted values of respect that are instilled from an early age.

*"Before a professor, all you can do is bow your head. That’s what I’ve been taught my whole life. Adults and professors-they're always to be respected. So, when a professor says something to me, I’ll never react negatively. I’ll never have an aggressive or defensive attitude, because I’ve always been taught to keep my head down." (E3)*

*.* Disregard is recognized as the act of not paying attention to comments, actions, or any stimulus to avoid being emotionally affected by these actions.

*"I don’t know if it counts as a defense mechanism, but ignoring the comment or action is what I do, so it doesn’t affect me. I mean, why would I pay attention to something that’s only going to affect me?" (E8)*

### Empowerment strategies

Reclamation: With this strategy, students not only defend themselves from harm, but also reaffirm their identity in a powerful and self-determined way. Through reclamation, the student intentionally takes negative words or attitudes and transforms them into something positive or neutral.

*"I’ve learned a method where I appropriate the insults. So, if someone calls me 'faggot,' I respond with, 'Yes, I’m a fag, I’m a poof.' It helps me not get hurt. For example, if someone calls me 'faggot,' I just say, 'Yeah, I am. So what?' I’ve gotten to the point where I don’t care. I appropriate the insult and say, 'Yes, that’s who I am, and I don’t care what you think.' It’s the best defense you can have." (E6)*

### University experience

#### Space of freedom

For the students interviewed, the university becomes a safe haven and a space of freedom:

*"I think of it as... I call it my escape place." (E3) and the opportunity for self-acceptance and free expression as expressed here "University, in general, has been very liberating. Here, the university allows you to discover yourself, without being stuck in the ideas you had before or what you were taught at home. It broadens your perspective. As for the LGBTQIA+ community, you feel accepted, whether you're attracted to men or women. Overall, university opens doors for you to understand, accept, and love yourself, and to express it freely." (E4)* This inclusive climate allows students to be open to new experiences: *"Socially, let's say that I hadn't really allowed myself to live openly as a homosexual person until I started university in my first semester. I felt like I could be myself and start fresh, almost like when you move to a new country and begin a new life. I said, 'I can be myself.'" (E5)*

#### Personal growth

Beyond liberation and acceptance, the university experience fosters significant personal growth and a broadening of perspective for nursing students:

*"It’s been a really enriching experience I mean*

*,*

*I’ve grown a lot as a person and in my way of thinking. I can definitely consider this as one of the best stages of my life. First, because I’ve fully accepted myself for who I am. And second, because I’ve learned to understand the uniqueness of other people- my feeling is different from the other person’s, we come from different backgrounds, and even our cultures are different." (E6).*

## Discussion

The university stage is a significant transition period for many young people, characterized not only by academic learning but also by personal development and the exploration of sexuality^20^. For nursing students, the academic journey from entry to obtaining their bachelor's degree involves interactions with stimuli from an environment that can be both enriching and unfavorable, particularly for those whose sexual orientation and gender identity differ from social norms and expectations. In this sense, this study sought to deepen the understanding of nursing students' experiences of stigma and discrimination based on their sexual orientation or gender identity. Three categories were identified: Stigma and discrimination, coping strategies, and university experience.

Ponce and Villanueva^2^ assert that discrimination against LGBTQIA+ individuals in university classrooms manifests in behaviors such as assuming everyone is heterosexual, using sexual orientation-related words as insults, making jokes, ignoring both the subject and individuals, justifying aggression, and blaming victims for being too visible. The results of this study indicate that nursing students encounter many of these forms of discrimination both in the classroom and at their practice sites. Additionally, they experience other forms of discrimination, including nonverbal language, such as dismissive looks and gestures of displeasure. The latter takes the form of microaggressions^21^ that convey the message that minorities are not welcome.

The findings of this study align with those of another, which indicated that while sexually diverse students acknowledge the university as a safe and reliable environment for expressing and recognizing sexual diversity, they still encounter patterns of stigma and discrimination. These issues exist not only on campus but also in their practicum settings, leading to a sense of discomfort among them^22^. Acceptance is not absolute, as some instructors occasionally respond with disapproving looks and make offensive, prejudiced comments toward individuals from the LGBTQIA+ community^22^.

Aguayo and Piña^23^ note that sexually diverse individuals are often frowned upon by society due to their preferences, opinions, and manner of dress. According to these authors, Latin American literature on homophobia suggests that while homophobia persists among higher education students, its manifestations have changed. Physical and verbal violence that infringed upon the rights of diverse individuals for being different is no longer predominant; instead, it has become covert, implicit, subtle, and often invisible to the casual observer^23^. However, the authors conclude that explicit discrimination persists. For instance, homosexuals are only tolerated if they are discreet or if they express themselves in specific spaces^23^.

It is important to note that nursing has historically been associated with a feminine connotation regarding caregiving^24^. This gender connotation may explain why the university community and care recipients exhibit stigmatizing and discriminatory behaviors toward nursing students who, upon entering the program, challenge social norms of what is considered 'normal.' Consequently, their ability to be, know, and perform in nursing is undervalued, as if a personal sexual identity diminishes their professional role performance. They are even questioned about how their identity might affect their nursing care practice.

It was found that the coping strategies used by the students in this study when faced with potentially discriminatory or stigmatizing situations ranged from adaptive to maladaptive, with the latter being predominant. Self-protective (maladaptive) strategies, such as concealment, submission, and disregard, may offer temporary relief but often lead to long-term consequences like isolation and alienation^25^. On the other hand, the adaptive strategy of reclaiming insults has proven effective in counteracting stigmatizing labels against sexual and gender minorities^26^.

Regarding the university, the findings ofthis study indicate that, in addition to being a place where competencies, skills, and abilities in a specific discipline are acquired, it also serves as a space for personal development and acceptance of gender identity. The perception of the university as a 'place of escape' and a 'space of freedom' highlights its crucial role in offering a safe environment where students can distance themselves from social pressures, explore their identities freely, and embrace their sexual orientation. In this regard, the university must create a protective, stable, inclusive, and pleasing educational environment for all students. This environment not only allows students to explore and express their identities while feeling supported but also maximizes their learning opportunities, ensures the achievement of academic goals^27^, and enhances their mental health and well-being^28^. On the other hand, the personal growth and expanded perspectives highlighted in the personal growth category emphasize that the university experience goes beyond academia, significantly shaping students' worldviews and social interactions. This aspect is particularly important in nursing education programs, where empathy and understanding are crucial for delivering human caring that individuals need, while also fostering human satisfaction in those receiving care^29^.

This study brings to light the complexity of the experiences faced by nursing students during their training, particularly concerning their sexual orientation and gender identity. However, it is important to note that the limitations of this study include the small sample size and the fact that the findings are based on statements from nursing students at a specific university. These factors suggest that extrapolating the results to other groups of nursing students should be cautiously approached. Additionally, it would be prudent to consider cultural, geographic, and institutional policy differences when interpreting these results. Despite these limitations, the results obtained constitute a valuable foundation for promoting interventions that not only support sexually diverse students but also collectively address stigma and discrimination to create truly inclusive academic environments. This includes the integration of sexual and gender diversity education into the nursing curriculum, the implementation of awareness and education programs for faculty, staff, and other university personnel, and the creation of an environment of respect and support for all students, regardless of sexual orientation or gender identity.

Future research exploring faculty and university administrative staff perceptions of sexual and gender diversity is needed, given their crucial role in fostering an inclusive educational environment. In this regard, such research should also include health professionals and workers from other fields who interact with nursing students at practicum sites, as these individuals significantly influence students' training and the quality oftheir academic experiences. This approach would help identify attitudes and practices that either reinforce or counteract stigma and discrimination, which is crucial for proposing and implementing effective, sustainable strategies that promote inclusion and respect in all professional educational settings. It is recommended that the experiences of stigma and discrimination among students from various fields of knowledge be explored to gain a deeper understanding of this phenomenon within the context of higher education.

## Conclusion

This study offered insights into the experiences of stigma and discrimination faced by nursing students based on their sexual orientation or gender identity during their academic training. It was found that nursing students with diverse sexual orientations face stigma and discrimination not only in the classroom from their professors but also at practice sites from care workers and care recipients. To cope with these situations, they use a range of strategies, from concealing their identity to reclaiming insults. Additionally, the university is seen as a meaningful space of liberation and acceptance, perceived as a safe environment where students can express themselves and experience significant personal growth.
